# Exploring modifications to rapid response systems in Norwegian hospital units

**DOI:** 10.1186/s43058-025-00817-7

**Published:** 2025-11-24

**Authors:** Jonas Torp Ohlsen, Miriam Hartveit, Stig Harthug, Marte Johanne Tangeraas Hansen, Siri Lerstøl Olsen, Hilde Valen Wæhle

**Affiliations:** 1 Department of Anesthesia and Intensive Care, Haukeland University Hospital, Bergen, Norway; 2 Department of Global Public Health and Primary Care, University of Bergen, Bergen, Norway; 3 Department of Research and Innovation, Fonna Hospital Trust, Haugesund, Norway; 4 Department of Clinical Science, University of Bergen, Bergen, Norway; 5 Stavanger University Hospital, Stavanger, Norway; 6 Department of Emergency Medicine, Stavanger University Hospital, Stavanger, Norway; 7 Department of Research and Development, Haukeland University Hospital, Bergen, Norway

**Keywords:** Rapid response systems (RRS), Early warning scores (EWS), FRAME, Implementation, Adaptation, Modification, Fidelity

## Abstract

**Background:**

Modifications and adaptations to evidence-based interventions are common, and of special relevance to complex interventions in healthcare. Although they play an important role in scale-up and sustainment, the potential exists for negatively affecting the core functions of an intervention. This study explores modifications to rapid response systems (RRSs), using the established Framework for Reporting Adaptations and Modifications – Expanded (FRAME). RRSs are patient safety interventions developed to identify and respond to hospital patients in clinical deterioration. Despite widespread use, little evidence-based guidance exists for necessary adaptations to local context. Applying adaptation frameworks is a novel perspective to improve RRS intervention design and implementation guidance. We aimed to explore which modifications and adaptations to RRSs that have taken place in Norwegian hospital units, how they occurred, and what the underlying reasons were by using FRAME.

**Methods:**

Nine hospital units across six hospitals, which had initiated the implementation of RRSs 4 to 12 years previously, were included. Data was collected through focus group and individual interviews with clinicians and leaders. Analysis involved two steps: a conventional, inductive content analysis to identify and categorize modifications, followed by further characterization of these modifications through deductive analysis employing FRAME.

**Results:**

Inductive analysis identified 5 categories and 24 subcategories of modifications to the RRS intervention. Application of FRAME revealed modifications to be mainly reactive and occurring in the maintenance/sustainment phase, decided at the unit level and with varying fidelity consistency. Both structured and informal processes were identified. The goals of modifications were improvement of feasibility, effectiveness and fit, and reasons were related to available resources, service structure, clinical judgment and patient factors. Minor adaptations to FRAME were necessary to fit the RRS intervention and the methods of data collection.

**Conclusions:**

Studying real-life implementations of RRSs provides insight in modification processes, highlights which intervention elements are modified for better fit and feasibility, and which modifications are prone to fidelity inconsistency. Our findings underline the ubiquity of modifications to RRSs, and the need to systematically anticipate them throughout all implementation stages. Further exploration of RRS core functions and application of FRAME within collectively implemented patient safety interventions could advance the field.

**Supplementary Information:**

The online version contains supplementary material available at 10.1186/s43058-025-00817-7.

Contributions to the literature

- This study characterizes modifications in real-life RRS implementation cases, presenting findings that are directly applicable for improvement of the intervention, as well as of RRS implementation and adaptation processes.
- We demonstrate the retrospective applicability of FRAME to RRS, but highlight issues related to defining single modifications, nature of content modification and fidelity consistency. Application of FRAME to collectively implemented patient safety interventions should be further explored.
- Based on our findings, the application of implementation science to RRS research could be advanced by further explorations of RRS core functions, their relative importance, and improved concepts of fidelity measurement.

## Background

Implementation of evidence-based interventions commonly lead to changes in the intervention itself [1]. These changes often represent efforts to enable feasibility and align the intervention to the local setting and contextual factors [2]. Regarded by some as unavoidable, there is an increasing understanding that these could play an essential role in scale-up and sustainment of an intervention [3]. However, to retain any proven effects on patient or user outcomes, a balance needs to be found between altering the intervention and preserving its core elements, the latter commonly conceptualized as fidelity, or the delivery of the intervention “as intended” [4]. In a terminology proposed by Stirman and used throughout this paper, a distinction is made between the terms *modifications* and *adaptations*: modifications refer to all changes to an intervention, including unplanned or reactive ones, while adaptations refer to a subset of modifications that are deliberate, pro-active, and more likely to be fidelity consistent [5, 6]. Adaptations are thought to be of special relevance to complex interventions in healthcare [7], where rigid implementation of protocols could be a barrier to uptake [8]. The study of adaptations is a developing field within implementation science with the potential to improve interventions, their implementation and ultimately patient-related outcomes [9–11]. Among a limited number of frameworks developed for reporting and describing modifications, a widely cited contribution is the Framework for Reporting Adaptations and Modifications – Expanded (FRAME) [5]. FRAME enables systematic, comprehensive descriptions of modifications, and has been applied to interventions from a diversity of fields [12–14].

### The intervention: rapid response systems

This study explores modifications to rapid response systems (RRSs), a patient safety intervention developed to identify and respond to hospital patients in clinical deterioration. An RRS has been defined as “a whole system for providing a safety net for patients who suddenly become critically ill and have a mismatch of needs and resources” [15]. It typically encompasses the use of an early warning score (EWS) for systematically observing vital parameters, defined thresholds for escalating care and a team-based response model. Different staffing models exist, often including nurses or physicians with critical care competencies in the response team [16].

There is increasing evidence that RRSs reduce hospital mortality and cardiac arrests [17]. Other reported effects have ranged from increased staff satisfaction [18] to increased workload for critical care staff [19], while effects on length of stay, intensive care admission and health economy are uncertain [17]. Although the intervention is widely recommended, major challenges remain for implementation, scale-up and sustainment [17, 20–22]. The literature recommends adaptation of several RRS elements to local context, such as choice of EWS or response model [16]. However, the relative effect of different models on RRS outcomes is unclear, and outcome studies often report sparsely on modifications to the intervention and other implementation aspects [23–25]. Limited evidence thus exists to guide such adaptation processes. Applying adaptation frameworks to studies of adult RRSs has not been reported previously and provides a novel perspective to inform and develop intervention design and adaptation guidance.

### Aims

The aim of the current study is to investigate local RRS modifications in Norwegian hospital units, using FRAME. Specifically, we wish to explore:

1. What modifications and adaptations have taken place?
2. How did they occur?
3. What were the underlying reasons?

## Methods

We employed a qualitative design, combining focus groups and individual interviews, to explore the units’ current RRS practice and, retrospectively, the modification processes leading up to these practices, as they had unfolded in a naturalistic setting.

### Setting

The study was conducted in six hospitals, all subsidiaries of the Western Norway Regional Health Authority, that had initiated the implementation of RRSs 4 to 12 years previously and were thus considered in the sustainment phase. Implementation efforts took place at the discretion of each hospital, not as part of the current study. In Norway, RRSs were initially recommended as part of a national patient safety campaign, and are now encompassed by separate national recommendations from the Directorate of Health [26]. These recommendations provide broad guidance encompassing both community health services and hospitals, focusing on a systematic approach to detection of deterioration and response. Although no specific RRS model is mandated, the National Early Warning Score 2 (NEWS2) and response algorithm is recommended [27]. Guidance discourages modifications to the NEWS2 score, escalation thresholds and observation intervals, while local adaptation of the response model is encouraged. Different models are in use throughout the region, and large variation between and within hospitals has been noted by the authors. A common electronic health record system is in use in the study setting, automatically calculating the NEWS2 from entered values.

### Participants: hospital units and informants

We included nine individual hospital units, employing purposive sampling of units, aiming to achieve diversity regarding hospital size, specialty of the unit, the presence of rapid response teams (RRTs) staffed by critical care personnel and possible differences in implementation trajectories. At the time of data collection, the median time from first RRS implementation in the unit was 5 years (range 4–12). Other characteristics of the participating units and hospitals are summarized in Table 1. Surgical units were abdominal surgery or mixed orthopedic/general surgical wards. Medical units were general internal medicine, gastroenterology or pulmonology wards. Selection of informants for focus group interviews was facilitated by contact persons and unit leaders at the relevant hospitals, and aimed to include both physicians and nurses, junior and senior staff, staff with experience of the implementation process, and staff representing the response team (if applicable). A total of 52 informants participated, representing the following professions: senior physicians (*n* = 11), junior physicians (*n* = 9), nurses (*n* = 19), nurse specialists (*n* = 7) unit leaders (*n* = 5) and quality advisor/simulation coordinator(*n *= 1). Reported work experience in current position and unit (available for *n *= 49 informants) was 1 to 38 years, median = 5.

**Table 1:** Characteristics of participating hospitals and units

Hospital	Bed capacity*	RRS first implemented	RRT available in hospital?	Unit designation	Unit specialty	Number of informants
1	138	2019	Yes			*n* = 7
				A	Surgical	
2	230	2020	Yes			*n* = 7
				B	Medical	
3	795	2018	No			*n* = 12
				C	Surgical	
				D	Medical	
4	78	2020	No			*n* = 5
				E	Surgical	
5	524	2012	Yes			*n* = 12
				F	Surgical	
				G	Medical	
6	59	2019	Yes			*n* = 9
				H	Surgical	
				I	Medical	

*RRT* Rapid Response Team (including critical care competent nurse or physician)

^*^Averaged, staffed bed availability for 2023

### Data collection

We aimed to conduct multidisciplinary focus group interviews to ensure a variety of professional viewpoints. If this diversity was not possible to achieve in one focus group interview, supplementary individual interviews were conducted. Additionally, a snowballing approach was allowed, to include individual interviews with relevant key informants brought to our attention by staff or contact persons. A semi-structured interview guide was developed, piloted and used for all interviews. Interviews were performed face-to-face in the hospitals by 1–3 researchers (JTO, HWV, SH) between November 2023 and June 2024, digitally recorded, and transcribed verbatim by the researchers. Names of respondents were anonymized. Interviewers had health professional backgrounds (physician or nurse specialist) and personal experience with RRS implementation.

### Data analysis

Analysis was performed in two sequential stages. First, we applied an inductive approach guided by conventional content analysis as outlined by Hsieh and Shannon [28]. Secondly, we employed a directed content analysis guided by the FRAME framework to further structure and interpret the data [5]. NVivo software (Lumivero, version 14, release 1.7.1) was used. The unit of analysis was the individual hospital unit or ward.

In the first step, two researchers (JTO, HVW) separately coded three initial interviews inductively, comparing results to ensure consistent and coherent analysis. Further coding then identified descriptions of modifications to the intervention that were grouped into subcategories in an iterative process. Collectively accepted ways of working that represented changes to the recommended intervention (NEWS2 2017 algorithm, including clinical response) were perceived as modifications. Where it was necessary to distinguish modifications from variations in fidelity, we emphasized the intention and motivation to adhere to the intervention or elements of it. For example, acknowledging that unwanted variation sometimes occurred, e.g. “we score according to the algorithm, but it is not always possible” would not be coded as modification. Further, all subcategories, representing groups of similar modifications, were abstracted into categories with accompanying descriptions that were discussed for consensus among all authors. To account for coding drift, all interviews were reviewed and re-coded if necessary.

In the second step of the analysis, all identified modifications were analyzed using FRAME. This analytical step involved assessing and classifying modifications against FRAME’s predefined categories, thereby enabling a detailed and systematic description of when and how modifications occurred, what was modified, by whom, underlying goals and reasons, and the relation to fidelity. For each hospital unit, we reviewed data from the modification subcategories, and coded the FRAME elements, following available online guidance, using Microsoft Excel (Microsoft 365, version 2408) [29]. Where data was missing to characterize modifications according to FRAME elements, this was noted. Following consensus discussions on the fit and validity of the framework application, coding was adapted to the current study for certain FRAME categories (nature of content modification, goal of modification and fidelity consistency). Fidelity was evaluated against the Norwegian national recommendations, that recommends NEWS2 but allows for local adaptation of the clinical response. A detailed list of adaptations to FRAME and their rationale is available as Additional File 1.

### Ethics and reporting

The study protocol was reviewed by the Regional Committee for Medical and Health Research Ethics (application 546242) and found exempt from regulations in the Health Research Act. The study was registered with and evaluated by the local Data Protection Officer, and all interview informants gave informed consent to participate. The study is reported using the consolidated criteria for reporting qualitative research (COREQ) as guidance (Additional File 2) [30].

## Results

A total of ten focus group interviews and eight individual interviews were analyzed. Median duration of interviews was 54 min (range: 33–67 min). The modifications identified are presented in the following and described using FRAME elements.

### Modification categories and related FRAME elements

The inductive part of our analysis identified 24 modification subcategories abstracted into five categories: *patient observation*, *triggers of escalation*, *responses to deterioration, response team workflows* and *communication around escalation. *Table 2 provides an overview of the categories, their associated subcategories and the nature of content modifications. Fidelity consistency is described in detail where dichotomous categorization was not possible. FRAME elements relating to the process and reasons of modifications are summarized per category below, and sample quotes are provided. A detailed overview of all available FRAME data and occurrence of the modification subcategories in the different units is available as Additional File 3.

**Table 2:** Modification categories, subcategories (n = units associated with each subcategory), descriptions and selected FRAME elements

**Category**	**Subcategory**	**Description** (bullet points = AND/OR unless specified)	**Nature of content modification** and **relation to fidelity** (adapted from FRAME)
Patient observation	Scoring on arrival in ward (*n* = 3)	Routinely scoring vital parameters shortly after patients arrive in the ward, such as when transferred from the emergency department or post-operative unit	Tweaking/refining by adding minor element to intervention. Fidelity consistent
	Exemption of patients with treatment limitations or in palliative care (*n* = 2)	Specific patients in palliative care or with treatment limitations (e.g., not candidates for intensive care) are exempted from NEWS2 scoring, but vital signs may be taken without calculating a score	Tweaking for patient categories that do not benefit from of the intervention. Fidelity consistent
	Individualized observation frequency (*n* = 8)	• Observation frequency determined on a case-by-case basis, and prescribed in fixed intervals (e.g., 2-hourly, 6 × per day), as a general strategy or for selected patients • Specific observation regimens for certain diagnoses (gastrointestinal bleeding, sepsis, pancreatitis) This may entail both more and less frequent observations than indicated by the NEWS2 algorithm	Combining with other observation regimens is fidelity consistent. Fixed interval observations remove the function of matching frequency to score severity, thus fidelity inconsistent
	Decreased observation frequency (*n* = 3)	Observation frequency for elevated scores decreased compared to NEWS2 algorithm, e.g. 8-hourly for NEWS2 1–4 or not continuously monitoring for NEWS2 ≥ 7	Reduces sensitivity of the system without removing the function completely. Fidelity inconsistent
Triggers of escalation	Use of changes in NEWS value (*n* = 5)	Using *changes* and the *magnitude of changes* in NEWS2 to decide: • when to escalate (nurses) • whether a clinical assessment is needed and how urgently (physicians)	Depending on specific circumstances this substitutes or adds a trigger mechanism and modifies the response. Certain applications may be fidelity consistent, but most cases are fidelity inconsistent
	Specification to RRT activation criteria (*n* = 1)	Specifying obligatoriness (recommended vs. mandatory) and maximum timeframe for attempts at stabilization in ward before calling RRT	Refinement of activation criteria. Fidelity consistent
	Reduction of NEWS2 for individual patients (*n* = 2)	Physicians exempting specific vital signs from the NEWS2 score for individual patients, generating a lower score	Ad-hoc modification to the scoring system in specific cases. Tweaking/tailoring that also reduces the sensitivity of the intervention, affecting a core function. Fidelity inconsistent
	Nurses’ individual judgment (*n* = 6)	Nurses assessing escalation on a case-by-case basis, emphasizing clinical and/or practical aspects of the situation that may outweigh the NEWS2 score Note: Using clinical judgment to escalate despite a *low* NEWS2 is not regarded as adaptation (encouraged in original intervention)	Substitutes or combines the trigger mechanism with individual situational and medical judgment. Some examples represent fidelity-consistent tweaking, while substituting defined call criteria with individual evaluation is fidelity inconsistent
	Increased threshold value (*n* = 2)	Increased general threshold for escalation to a physician, such as NEWS2 7 (red) rather than NEWS2 5 (orange)	Systematically increasing the trigger level, thus lowering the sensitivity of the EWS. Fidelity inconsistent
	Acceptance of high scores (*n* = 9)	Accepting scores above escalation threshold without escalation, due to: • deviating vitals attributed to pre-existing medical conditions • persistence over time • physicians’ evaluation that score is acceptable without escalation	Some examples of tweaking using clinical reasoning, but all modifications lower EWS sensitivity. Decisions made for single patients and documented by physician may be fidelity consistent, while accepting high scores only due to their persistence over time is not
Responses to deterioration	Ward-based response post-RRT pilot (*n* = 2)	Using a ward-based response model after initial piloting of ICU-staffed RRT	The RRT was removed after initial piloting and replaced with a ward-based response. Fidelity consistent
	Use of cardiac arrest team (*n* = 3)	Cardiac arrest (CA) team used for severely ill (non-CA) patients when instant response needed and not delivered by local RRT or ward-based response team	Tailoring/tweaking when used ad-hoc in rare circumstances; Adding or substituting element from a systems perspective. Fidelity consistent
	Graded response within department (*n* = 5)	• On-call physician assessing degree of urgency, need for bedside evaluation and/or initiating measures by phone • Assessment by the department’s senior on-call physician before escalating to RRT, to assess if patient can be handled in the departments’ own intermediate care unit	Adaptation to local service structure is tailoring and fidelity consistent. Remote re-evaluation of escalations by on-call physician could alter the core function of having an escalation trigger, but may be fidelity consistent if part of defined local practice
	Preservation of established response practices (*n* = 2)	Physicians perceive NEWS as solely a nursing tool for deciding when to contact the rounding or on-call physician, and respond to calls about deteriorating patients in the same way(s) they did before RRS implementation	Skipping or removing the core function of a protocolized response. Fidelity inconsistent
	Nurse-initiated measures (*n* = 8)	Diagnostic or treatment measures initiated by nurses in response to elevated EWS score, prior to or concurrent with escalation to a physician	Tailoring/tweaking to local context by adding or substituting elements (nurse-driven response substitutes physician's review). Fidelity consistency is mixed, depending on whether the nurse-initiated measures substitute and/or postpone escalation (inconsistent), or are concurrent to escalation (consistent)
	Bypassing RRT (*n* = 5)	Staff are aware that the option of convening an RRT exists but mount a response through other pathways, such as direct or informal contact between ward staff and senior/ICU physicians	Mainly removing or skipping element, but some examples of well-argued bypassing of RRT for individual cases represent tweaking. Fidelity consistency is accordingly mixed, but mostly inconsistent
Response team workflows	Team composition (*n* = 3)	Composition of RRT in terms of staff, defined locally at the hospital level; may vary according to time of day to match what HCWs are available on-call	Tailoring, fidelity consistent
	Tailored selection of response staff (*n* = 1)	RRT HCWs having the option of case-by-case decisions on what staff is deployed from the ICU when they are contacted by ward personnel	Tweaking/tailoring/refining, fidelity consistent
	Standardized RRT medical equipment (*n *= 3)	Standardized medical equipment in a backpack used by the RRT when deployed	Refining, fidelity consistent
	RRT as ward resource substitute (*n* = 2)	RRT personnel substituting for ward staff to facilitate timely clinical review and/or treatment. Examples: ward nurses not available to monitor patient during transport or intervention, on-call surgeon in operation and not available to review patient	Adding an element to the original scope of the RRT. Fidelity consistent
	Proactive RRT nurse rounding (*n* = 1)	ICU nurse on RRT duty proactively rounding the wards, to establish liaison and identify potential cases for RRT activation or ICU transfer, if workload allows	Adding an element to the original scope of the RRT. Fidelity consistent
Communication around escalation	Standard RRT communication form (*n* = 1)	Form used by RRT nurses to standardize how to receive and respond to a call for RRT activation	Refining in specific context. Fidelity consistent
	Staff accepted to activate RRT (*n* = 3)	Both nurses and physicians can activate the RRS	Tweaking/tailoring/refining. Fidelity consistent
	Clinical concern outweighing EWS (*n* = 3)	Nurses emphasize clinical concern in addition to NEWS2 value to obtain an appropriate response for values above escalation threshold. Physicians emphasize such concern, in addition to the professional experience of and/or their personal knowledge of the nurse initiating the escalation	Modifies escalation mechanism by incorporating or substituting clinical concern and interpersonal factors. Escalating on clinical concern alone is fidelity consistent; other uses described are fidelity inconsistent

*FRAME* The Framework for Reporting Adaptations and Modifications – Expanded, *NEWS2* National Early Warning Score 2, *EWS* Early warning score, *RRT* Rapid response team, *ICU* Intensive care unit, *HCW* Health care worker, *Tweaking* Minor changes addressing immediate and concrete concerns, *Refinement* Attempts at optimizing functions of intervention, *Tailoring* Changes to ensure relevance to specific context

#### Patient observation

Modifications mainly took place in the maintenance/sustainment phase; only one unit had one modification in the implementation phase. The process, if described, comprised both planned, reactive adaptations and unplanned modifications. Decisions were made by the organizational unit/team or, in one instance, a coalition of stakeholders. Goals were to improve feasibility, fit with recipients (patients) and effectiveness, based on reasons related to available resources at the organizational level, clinical judgment at the provider level, and comorbidity and current diagnosis at the patient level.

“We’ve made a few changes, local adaptations. […] We formed a small group to discuss it. The thing is, when you have to implement something, it has to feel useful for people to adhere to it. And with a score of five, you have to measure at least once per hour. And we see that very many patients have a score of five, often because they are postoperative cases. Elderly patients, often with underlying COPD….it doesn’t take a lot to pass a NEWS of five. If we then have to measure once per hour and call the doctor, and the doctors on their side signal that we’re calling too often… So, for us to adhere to the procedure we had to feel that we DID get help, and that it was useful to follow it. So, we agreed on changing it to every three hours, and to call if there’s clinical deterioration.” (Nurse)

#### Triggers of escalation

Modifications took place in the maintenance/sustainment phase. The process, if described, comprised both planned, reactive adaptations and unplanned modifications. Decisions were made by the organizational unit/team or a coalition of stakeholders. Goals were to improve feasibility, fit with recipients (patients), fit with context and effectiveness, as well as increasing reach or engagement. Reasons for modifications related to existing policies at the sociopolitical level, social context (one subcategory) and available resources at the organizational level, clinical judgment at the provider level, and comorbidity at the patient level.

“It also makes it easier for the nurses not to have to sound the alarm all the time. At least in our department, with our patient group….if we had strictly followed the criteria, I think it would have created a fatigue in the system that eventually would make us unable to deal with it. Now we feel that we have a tool that we actually trust and use..." (Physician)

#### Responses to deterioration

Modifications took place in the maintenance/sustainment phase, and were both planned, reactive adaptations and unplanned modifications. Where described, decisions were made by administrators and the organizational unit/team. However, data on the decision-makers were not available for most of the subcategories. Goals were to improve feasibility, effectiveness and contextual fit. Reasons for modifications related to available resources, service structure and social context at the organizational level, and clinical judgment and previous training and skills at the provider level.

“So, there was this discussion if we should call the RRT when a patient is deteriorating. Our experience, which is all about the local context, is that the physical distances and lines of communication are short. […] Often, when I ask the intensivist “Do you want to join me and have a look at the patient, so we can decide if he should be transferred?” they answer, “just transfer him, I’ll have a look at him when I pass by the unit”. So, our logistics are much easier”. (Physician)

#### Response team workflows

Modifications took place in the maintenance/sustainment phase, except for one case of pre-implementation, proactive adaptation. The remaining modifications were both planned, reactive adaptations and unplanned modifications. Decisions were made by administrators, the organizational unit/team or a coalition of stakeholders. Goals were to improve contextual fit, effectiveness and reach/engagement. Reasons for modifications related to existing policies at the sociopolitical level, available resources, service structure and social context at the organizational level, and clinical judgment and perception of intervention at the provider level.

“What we’ve tried, to better get to know each other and improve collaboration between ICU and the wards, for some shifts, nurses have been tasked to round the hospital, wearing their vest saying “ICU nurse”, the one they’re wearing when they’re called out, and introduce themselves: “Hello, I’m the RRT nurse today, how are things going here and do you think you might need help with something?”. It makes us more harmless. The door to the ICU is locked, and very few people see the inside, so they don’t know. That’s something I don’t think we’ll ever avoid completely, but if we could just be seen as a resource, as a support rather than something intimidating. So, it’s been very positive”. (Unit leader)

#### Communication around escalation

Modifications took place in the maintenance/sustainment phase, and were both planned, reactive adaptation and unplanned modifications. Decisions were made by a coalition of stakeholders, if specified. Goals were to improve reach or engagement, feasibility and effectiveness, for reasons related to social context and available resources at the organizational level, and perception of the intervention and clinical judgment at the provider level.

Nurse A: “If we just call and say that the patient has a NEWS of seven, it happens that they’re very relaxed [the physicians]. We’re often told to wait and see. It’s not always they respond so quickly. Unless maybe if we are a bit pushy. And even then, not always.”

Nurse B: “We’ve definitely learned what to say to get a response”.

Nurse A: “Yes, over time. But if you haven’t learned it, it takes a little time. You have to know what to emphasize. And how to present it”.

Nurse B: “Yes, but you can’t exaggerate all the time, that’s wrong. Eventually you understand WHEN you really need the one on the other end to respond”.

Interviewer: “What is it you have to say, then?”

[Laughter]

Nurse A: “I usually say that I’m worried. About the patient. And that I personally perceive the patient as much worse than before”.

### Summary across categories

Individual units were associated with a median of 9 modification subcategories (range 5–15). The number of units associated with specific subcategories specified for medical specialty and hospital size is reported in Additional File 4.

Across categories, all units reported multiple modifications that were almost exclusively reactive and occurring during the maintenance/sustainment phase. Half of the modification subcategories were fidelity consistent, while the remainder was equally divided between fidelity inconsistent and a mixed relation to fidelity. Out of 16 the modification subcategories containing adaptations (planned reactive or proactive processes), 10 were fidelity consistent. Commonly decided at the unit level, processes were most often informal and not always well defined – “it just turned out that way”. Despite all units having multiple modifications, only 4 out of 9 units reported having structured adaptation processes. Improving feasibility was the most common goal, followed by improving effectiveness and fit to context and recipient (patients). Correspondingly, the most common reasons were available resources (almost exclusively referring to human resources) and service structure at the organizational level; clinical judgment the most common reason at provider level; and comorbidity and current diagnosis the most common reasons at the recipient (patient) level.

Regarding the specialty of the unit, on-call surgeons were reported to regularly be unavailable for escalation due to ongoing surgical procedures, resulting in modifications/adaptations that reinforced nurse autonomy in initiating measures or escalating directly to a senior clinician or RRT. Informants also characterized on-call physicians in medical wards as exhibiting a stronger presence in patient follow-up, and familiarity with typical clinical challenges associated with deterioration. However, in a large hospital, sub-specialization of internal medicine physicians was reported as a barrier to timely and comprehensive review of surgical ward patients with non-surgical deteriorations, which was cited as supporting the case for having an RRT. In a small hospital, short lines of communication and close work relations between staff were explanations for using informal escalation pathways, outside of the RRS; contact between surgeons and anesthesiologists/intensivists was facilitated by close working relations in the perioperative setting.

Minor variations in personal work routines between staff often surfaced in the interviews. In one unit, there was major divergence on descriptions of the work routines between nurses and physician (interviewed separately).

## Discussion

In this study, we have identified a multitude of modifications and adaptations to both afferent and efferent parts of the RRS, that were mainly reactive and with varying fidelity consistency. Both structured and informal modification processes were identified. The goals of the modifications were improvement of feasibility, effectiveness and fit, and their reasons were related to resources, service structure, clinical judgment and patient factors.

The modifications reported demonstrate how clinicians and unit leaders attempt to integrate the intervention into the existing processes of everyday clinical work and accommodate the constraints of limited time and human resources, within existing organizational structure and interprofessional and interdepartmental hierarchies. Often, clinical judgment or common sense is applied to align the intervention with their work setting, existing workflows and patient population. However, how this affects the effectiveness of the intervention remains unknown.

### Reasons for modifications and the relation to fidelity

We encountered many fidelity-inconsistent modifications, and a high prevalence of unplanned, reactive or absent processes. While fidelity challenges for RRS implementations are well described, there is sparse literature using the concept of modifications or adaptations. Our findings contrast with those reported in a survey of adaptations to a pediatric EWS intervention [14], that were found to be mostly fidelity consistent. This could relate to the longer sustainment times observed in our study, or other factors such as differences between supported and unsupported implementation trajectories. The choice of methodology has also been shown to affect the descriptions obtained [13]. Compared to surveys, one could speculate that interviews would identify more informal modifications that are prone to being fidelity inconsistent. In our study, the goal of modifications was often to improve feasibility. Clinicians described how not having the resources (staff, time) to adhere to the original intervention protocol, or being measured against a standard perceived as impossible to achieve, led to de-motivation. Ensuing modifications often sacrificed intervention fidelity to achieve better feasibility and subsequently motivation and engagement. This highlights the dilemma of how to balance adaptations against fidelity, and indicates poor fit of the un-adapted intervention. Frameworks and models for prospective adaptation decision-making, such as IDEA (Iterative Decision-making for Evaluation of Adaptations), ADAPT or MADI (Model for Implementation Design and Impact), would in theory be helpful in such a situation [31–33]. Apart from the acknowledged difficulties of applying such tools outside of a research settings, challenges to their application to RRSs are the lack of consensus on well-defined core functions and/or intervention outcomes that can readily be measured during a pilot. Core elements or functions, defined as those elements or functions of an intervention that are efficient and necessary [34], is an underexplored concept for RRSs. Although one commonly describes *afferent* and *efferent* limbs as key parts of an RRS, using terms that imply certain functions, explorations of how they are connected or their sub-elements is rarely encountered [8, 16]. Related to this discussion, one could question whether a dichotomous classification of the relation to fidelity offers sufficient nuance. For the current study, it proved challenging to apply during analysis. Multiple dimensions of fidelity have been outlined, in implementation theory and evaluations [4, 35]. Additionally, although RRSs can be envisioned as a linear chain of events [36], hinting at equal importance of all the “links” (or equal necessity of their presence), it is unlikely that all intervention elements carry equal importance. For instance, it has been proposed that the afferent limb provides most of the clinical effect compared to other RRS elements [16].

Informal modification processes seem to find the “path of least resistance”, and may lose focus on the overall goal, function or “spirit” of the intervention. They may be especially relevant for collectively implemented interventions, where multiple providers are involved in different parts of the intervention, at different times. Additionally, situations involving mandated implementation could amplify such mechanisms if implementation is distorted into a tick-box exercise. Among the fidelity-inconsistent modifications found in our study, we find that the ones most likely to be detrimental to RRS effectiveness are those that weaken the function of RRS as a *system* (e.g. subcategories of *preservation of established response practices*, and *bypassing RRT),* and those that potentially delay an adequate response (e.g. category of *triggers of escalation*).

### Learning from modifications

An important goal of reporting and studying modifications and adaptations is to improve the intervention, its implementation and outcomes [9, 11]. While fidelity-consistent adaptations are theoretically optimal, even fidelity-inconsistent modifications could carry a potential for learning and refinement of the intervention [31]. The reasons for modifications may also point to contextual implementation factors that could be addressed through adapted strategies. Some examples from our study will be highlighted in the following sections.

A common modification was to reduce the frequency of observations. For higher NEWS2 scores, clinicians universally perceived it as difficult or impossible to achieve the recommended frequency in a standard ward, and transferring to higher levels of care rarely happened purely for observation. The NEWS2 guidance specifies that the frequency of monitoring *following an escalation* can be defined locally. However, that specific adaptation was rarely reported. The underlying constraints related to workload, staffing levels and availability of intensive care unit (ICU) beds need addressing at a systems level. However, while reducing the observation frequency is fidelity inconsistent, the evidence-base behind the recommended intervals is sparse [37], and modifications that preserve the concept of increasing observation frequency with increasing score are intuitively less detrimental than other modifications. Such considerations could be included in pragmatic adaptation guidance that reflect local resource availability.

For modifications on *triggers of escalation*, a central aspect was the need to adapt to patient groups with elevated NEWS2 scores due to pre-existing conditions, or those with persisting, high scores for other reasons. Although supplementary implementation guidance for NEWS2 stress that the trigger levels can be individualized on clinical grounds, there is evidence that such alterations are associated with worsened clinical outcomes without reducing the number of RRT calls [38, 39]. In some studies, only a minority of scores above the trigger threshold lead to an escalation [40]. This category thus represents modifications that are common and necessary, but have potential to negatively affect core functions. Clinically sound reasoning at the patient level may logically mitigate this. However, well-contemplated ad hoc decisions by individual clinicians require a thorough understanding of all aspects of the intervention and could represent a larger risk of changes to core functions than more systematic approaches. We did not encounter descriptions of individualized trigger thresholds; rather, the modifications we report handled the issue by reducing the score, emphasizing changes in NEWS2 value, using individual judgment or simply accepting elevated scores without specifying an alternative trigger value. This issue often concerned specific patient groups in the participating units. While NEWS2 was updated with adaptations for patients with hypercapnic respiratory failure, its explicit purpose is to have one standard EWS tool [27]. Contrasting this goal, numerous disease-specific EWS are available, for instance for respiratory disease [41], with potentially better fit. While we found several examples of pre-existing diagnosis-specific observation regimens used in combination with NEWS2, these were not EWSs and were employed to have more frequent and comprehensive observations than NEWS2. A potential solution to this issue may be individual risk stratification, already applied by machine-learning algorithms in some clinical settings [42]. Such models may however present their own problems, including the need for careful calibration to avoid alarm fatigue, and do not eliminate the need for individual clinical evaluation by a human [43]. An alternative model is the formal inclusion of individual clinical judgment in the EWS, a model that has been shown non-inferior to a standard EWS [44].

Half of modification subcategories were related to the response or efferent part of the RRS. All studied units were subject to national recommendations that allow complete local adaptation of the response model, requiring that it is defined, disseminated and provides a timely and competent medical response. Only two hospitals had RRTs with regular activations. Our findings in this category highlight difficulties in estimating actual needs or fit for context *prior* to implementation, pointing at the need for planned, iterative evaluation and adaptation. Such processes, identifying which adaptations could help overcome barriers, as well as evaluating their relation to core functions and prioritizing them for action, could draw on quality improvement tools already commonly employed with patient safety interventions [25]. This would also facilitate early identification of inappropriate modifications, or intervention elements trending toward abandonment, requiring reinforcement of activities or adaptations of the intervention to improve fit and feasibility. Local adaptation of the response model is encouraged in the literature [16], and RRS models employing ward-based responses may be valid [45, 46]. However, we observed a risk of continuing “business as usual”, where physicians regard the EWS purely as a nursing tool, and do not adopt the RRS concept in their clinical routines. This was related to gaps in interprofessional training/simulation (conducted as one-off events, without follow-up or trainings for new staff) and leadership support, shown to be important for RRS functioning [47]. A related theme on interprofessional aspects is the prevalent practice of ward nurses instigating stabilizing measures. Although empowering all staff to handle emergency cases within the limits of their professional competencies is encouraged nationally, these adaptations need to be considered carefully, so as not to delay or substitute timely escalation to competent providers. Finally, as an RRT typically covers an entire hospital, a complicating factor for this response model is the need for coordinating adaptations across departments and units, leaving less room for informal processes at the unit level. Hospitals that succeeded in having structuring cross-departmental adaptation processes, encountered in the current study, often used both qualitative and quantitative data on RRS function for feedback, and had well-defined organizational oversight of the RRS.

### FRAME application and fit

The FRAME was developed using studies of public and behavioral health interventions but has been applied to interventions in other fields [12–14]. We found the core concept of describing the “what, why, who, how and when” of modifications and adaptations both relevant and applicable to studies of RRS implementation. Still, minor adaptations were necessary (see Additional File 1), related to the specific intervention and the methodology of our study. We applied FRAME retrospectively and after a long period of sustainment, contrasting to prospective applications of FRAME within a shorter sustainment timeframe [12]. Conceptual sustainment literature emphasizes both the need for application of frameworks, and the importance of exploring sustainment determinants [48]. While it cannot achieve the same level of granularity as prospective application, we found that retrospective use of FRAME responds to this gap, offering insights into how adaptations or modifications, potentially perceived as “program drift” [11], may affect sustainment. However, fully characterizing these phenomena in this stage is empirically complex, highlighting the necessity of multiple methodological strategies to understand long-term sustainment. Our approach allowed nuanced descriptions of real-life practice but resulted in missing data for some FRAME elements. It also raised the question of how to define one single modification, when not defined directly by the respondents themselves, for which we did not find published guidance. Given the collective nature of delivery, a very large number of minor variations in practice could potentially be defined as individual modifications. Our approach was to apply FRAME at a compiled, subcategory level. While potentially sacrificing some solution in the results, this approach facilitated analysis and discussion. Some response options did not fit the RRS intervention well. For example, the “nature of context intervention” options seemed appropriate for an intervention delivered by single providers in discrete modules. By contrast, RRSs are complex, collective interventions, centered around dynamically applied functions, the concrete enactment of which will differ from case to case. Our solution was a narrative description, using FRAME terms where applicable. For stating the goals of the adaptation, most FRAME options were relevant, but we found the need for an option for improving fit to *context* (existing work culture and practices) rather than *recipients.* This likely reflects the nature of this type of intervention, in which the ultimate “recipients” are the patients, but the behavior change happens among health care workers. We could not categorize the relation to fidelity as consistent or inconsistent for all modifications and found detailed descriptions of mixed relations to fidelity more useful than using the FRAME option of “unknown”. Lastly, there is no universal, detailed RRS model against which to measure fidelity, and the concept of RRS core functions is not well developed in the literature. Therefore, each application of FRAME to RRS implementations must establish its own definitions, which reduces generalizability.

### Limitations

For limitations of the current study, we acknowledge that the descriptions of modifications per case may not be exhaustive with the applied methodology, and data on previous implementation efforts may be affected by recall biases and missing data due to staff turnover. Also, we do not have data to characterize fidelity in the units after initial implementation efforts, and if this changed over time. We note that units with ongoing or recent quality improvement measures or adaptation processes provided more detailed information, introducing potential bias. As descriptions of modifications sometimes diverged between professional groups, it may have affected our results that not all focus groups had a multidisciplinary representation which could have facilitated direct discussions of these divergences. We evaluated fidelity against an RRS model specifically using the original NEWS2 algorithm, for which the national recommendations did not allow general adaptations of observation intervals or escalation thresholds. Had such adaptations been permitted, fidelity consistency would likely have been higher. Although our findings may not necessarily be generalizable outside our study setting, many of the reasons found for modifications concurs with previously reported barriers and facilitators [49, 50].

## Conclusions

Studying real-life implementations of RRSs using an established framework revealed a multitude of modifications that were mostly unplanned, reactive and with varied relation to fidelity – only a minority of these can thus be termed adaptations. Our findings:

1. Provide important insight in how health care workers modify the intervention, highlights which intervention elements are modified to obtain fit and feasibility in specific contexts, and which modifications are prone to fidelity inconsistency.
2. Underline the necessity of anticipating adaptations in all implementation phases through a systematic approach, including iterative processes to provide structure for reactive adaptation.
3. Serve as indicators of where intervention design and pro-active adaptations should focus in future implementations or improvement projects.

Guidance for RRS implementation is needed that provide concrete and practical advice based on available evidence, frameworks and improved definitions of RRS core functions. More comprehensive evaluation of adaptation frameworks for collectively implemented patient safety interventions is warranted.

## Supplementary Information

Additional file 1. FRAME constructs and adaptations.

Additional file 2. COREQ checklist.

Additional file 3. FRAME data.

Additional file 4. distribution of modifications.

## Data Availability

A detailed summary of our analysis according to FRAME is provided in Additional File 3. The interview transcripts generated and analyzed in the current study are not publicly available due to risk of compromising individual confidentiality. Processed data can be made available (in Norwegian) from the corresponding author on reasonable request, and with permission of the Data Protection Officer at the relevant hospital.

## Abbreviations

EWS: Early warning score
FRAME: Framework for Reporting Adaptations and Modifications – Expanded
ICU: Intensive care unit
NEWS2: National Early Warning Score 2
RRS: Rapid response system
RRT: Rapid response team
