# Supplementary material for: Exploring modifications to rapid response systems in Norwegian hospital units
Source: Implement Sci Commun. 2025 Nov 24;6:129. doi: 10.1186/s43058-025-00817-7 (PMC12642216; doi:10.1186/s43058-025-00817-7)
Supplement: Supplementary file 1 — Additional file 1. FRAME constructs and adaptations. [file 43058_2025_817_MOESM1_ESM.docx]

FRAME constructs^[[1]](#endnote-1)^, annotated with adaptations for the current study and their rationale, in addition to specifications or comments

| **FRAME heading** | **FRAME elements and options** | **Adaptations and specifications for this study** |
| --- | --- | --- |
| **Process** | **When did the modification occur?**   - Pre-implementation/planning/pilot - Implementation - Scale up - Maintenance/Sustainment |  |
|  | **Were adaptations planned?**   - Planned/Proactive adaptation - Planned/Reactive adaptation - Unplanned/Reactive modification |  |
|  | **WHO participated in the decision to modify**   - Political leaders - Funder - Organizational unit/team - Tx developer/purveyor - Administrator(s) - Tx team - Provider - Program staff - Community members - Coalition - Recipient - Other________________ | Organizational unit: ward  Coalition: used if stakeholders such as patient/relative representatives were included e.g. in project group |
|  | ***WHAT* is modified?**   - Content - Context - Training and Evaluation - Implementation and scale-up activities | All adaptations/modifications were *content* in the current study |
|  | **Contextual modifications are made to which of the following?**   - Format - Setting - Personnel - Population | Not applicable for current study, see above |
|  | **At what *LEVEL OF DELIVERY* (for whom/what is the modification made?)**   - Individual - Target Intervention Group - Cohort - Individual practitioner - Clinic/unit level - Organization - Network System/Community | Organization specified to “hospital” |
|  | **What is the *NATURE* of the content modification?**   - Tailoring/tweaking/refining - Changes in packaging or materials - Adding/Removing/skipping elements - Shortening/condensing/Lengthening/ extending (pacing/timing) - Substituting/Reordering/Spreading of intervention modules or segments - Integrating parts of the intervention into another framework - Integrating another treatment into the intervention or practice - Repeating elements or modules - Loosening structure - Departing from the intervention (“drift”) followed by a return to protocol within the encounter - Drift from protocol without returning | Poor fit for the current study (irrelevant and/or overlapping options). Characterized by combined free text descriptions of adaptation subcategories and detailed description of the nature of the content modifcation. |
|  | **Relationship to fidelity/core functions?**   - Fidelity Consistent/Core elements or functions preserved - Fidelity Inconsistent/Core elements or functions changed - Unknown (can specify possible/likely consistent/inconsistent) | Challenging to unequivocally define in current study (see “methods” section). Characterized in detailed text descriptions for each adaptation subcategories. |
| **Rationale (Why?)** | **What was the goal?**   - Increase reach or engagement - Increase retention - Improve feasibility - Increase satisfaction - Reduce Disparities or Promote Equity - Improve fit with recipients - To address cultural factors - Improve effectiveness/outcomes - Reduce cost - Increase access | “Improve fit…” adapted to   - “Improve contextual fit” - “Improve fit to patients”   as two distinct common goal was improving fit to 1) the work context and 2) the unit’s specific patient groups |
|  | **Reasons: Sociopolitical**   - Existing Laws - Existing Mandates - Existing Policies - Existing Regulations - Political Climate - Funding Policies - Historical Content - Societal/Cultural Norms - Funding or Resource Allocation/ Availability - Stigma |  |
|  | **Reasons: Organization/Setting**   - Available resources (funds, staffing, technology, space) - Competing demands or mandates - Time constraints - Service structure - Location/accessibility - Regulatory/compliance - Billing constraints - Social context (culture, climate, leadership) - Mission - Cultural or religious - Identified disparities in services provided | For the studied intervention, the distinction between “available resources”, “time constraints” and “service structure” was not always clear. If doubt, “available resources” was used. |
|  | **Reasons: Provider**   - Race - Ethnicity - Sexual/gender identity - First/spoken languages - Previous Training and Skills - Preference - Clinical Judgment - Cultural norms, competency - Perception of intervention - Comfort with/availability of technology | Provider reasons were coded as referring to providers as a collective, not individual providers. |
|  | **Reasons: Recipient**   - Race; Ethnicity - Gender identity - Sexual Orientation - Access to resources - Cognitive capacity - Physical capacity - Literacy and education level - First/spoken languages - Legal status - Cultural or religious norms - Comorbidity/Multimorbidity - Immigration Status - Crisis or emergent circumstances - Motivation and readiness - Comfort with/availability of technology - Experience of discrimination, stigma - Mistrust of the system - Other______________________ | Recipient reasons were coded for patient related reasons, related to patients as a group. Only one was relevant to use, “comorbidity/multimorbidity”. In addition, “other: current diagnosis” was specified. |

1. FRAME 2023 codebook, accessed from: <https://www.med.stanford.edu/fastlab/research/adaptation.html> on 20.12.2024 [↑](#endnote-ref-1)
