# Supplementary material for: Exploring modifications to rapid response systems in Norwegian hospital units
Source: Implement Sci Commun. 2025 Nov 24;6:129. doi: 10.1186/s43058-025-00817-7 (PMC12642216; doi:10.1186/s43058-025-00817-7)
Supplement: Supplementary file 4 — Additional file 4. distribution of modifications. [file 43058_2025_817_MOESM4_ESM.docx]

Distribution of modifications/adaptations among units (N=9), specified by hospital size and medical specialty.
